# Design and Analysis of Gallium Arsenide-Based Nanowire Using Coupled Non-Equilibrium Green Function for RF Hybrid Applications

**DOI:** 10.3390/nano13060959

**Published:** 2023-03-07

**Authors:** Pattunnarajam Paramasivam, Naveenbalaji Gowthaman, Viranjay M. Srivastava

**Affiliations:** 1 Electronics and Communication Engineering, Prince Shri Venkateshwara Padmavathy Engineering College, Chennai 600127, India; 2 Department of Electronic Engineering, Howard College, University of KwaZulu-Natal, Durban 4041, South Africa

**Keywords:** nanowire, tight binding models, NEGF, GAA, hydrostatic strain, microelectronics, nanotechnology, VLSI

## Abstract

This research work uses sp^3^d^5^s* tight-binding models to design and analyze the structural properties of group IV and III-V oriented, rectangular Silicon (Si) and Gallium Arsenide (GaAs) Nanowires (NWs). The electrical characteristics of the NWs, which are shielded with Lanthanum Oxide (La_2_O_3_) material and the orientation with z [001] using the Non-Equilibrium Green Function (NEGF) method, have been analyzed. The electrical characteristics and the parameters for the multi-gate nanowires have been realized. A nanowire comprises a heavily doped n^+^ donor source and drains doping and n-donor doping at the channel. The specified nanowire has a gate length and channel length of 15 nm each, a source-drain device length L_SD_ = 35 nm, with La_2_O_3_ as 1 nm (gate dielectric oxide) each on the top and bottom of the core material (Si/GaAs). The Gate-All-Around (GAA) Si NW is superior with a high (I_ON_/I_OFF_ ratio) of 1.06 × 10^9^, and a low leakage current, or OFF current (I_OFF_), of 3.84 × 10^−14^ A. The measured values of the mid-channel conduction band energy (E_c_) and charge carrier density (ρ) at V_G_ = V_D_ = 0.5 V are −0.309 eV and 6.24 × 10^23^ C/cm^3^, respectively. The nanowires with hydrostatic strain have been determined by electrostatic integrity and increased mobility, making them a leading solution for upcoming technological nodes. The transverse dimensions of the rectangular nanowires with similar energy levels are realized and comparisons between Si and GaAs NWs have been performed.

## 1. Introduction

Over the last two decades in the semiconductor industry, the advanced structure of Metal Oxide Semiconductor Field Effect Transistors (MOSFETs), from planar to multi-gate design, has been proposed to achieve great electrostatic control over the channel. Various multi-gate structural designs named Double gate, Tri-gate, Pi, Omega, Top on-one side, and Gate-all-around (GAA) devices with nanotechnology approaches have been used for forthcoming applications. The GAA device architecture has high resistance properties. It exhibits static control on the gate over the conduction of the channel, which plays a major role in avoiding short-channel effects [1,2]. Natori et al. [3] have proposed GAA NW to resist (SCEs) by improving gate length and channel length scaling (L_G_ < 5 nm and L_ch_ < 15 nm). The primary benefit of GAA devices is that they have a higher I_ON_/I_OFF_ ratio [4]. The reduction in OFF current (I_OFF_) produces a high (I_ON_/I_OFF_) ratio. Batakala et al. [5] have demonstrated the comparison between Si and GaAs GAA MOSFETs. The gain and current-driving capacity of the GaAs material were efficient and, thus, based on this channel material, was selected following the application field. The reduction in leakage current had been achieved by considering major features, such as smaller threshold voltage (V_th_ ~0.3 V), channel length (L_ch_ ≥ 10 nm), shorter gate length, high dielectric constant oxide material, and the dopant concentration of the source, channel, and the drain, respectively [6].

Several studies have analyzed NWs with multi-gate arrangements. Wang et al. [7] reduced the computational complexity in SiNW by the scattering effects, and an approximation of the effective mass was carried out using Buttiker probes. Arun et al. [8] have proposed SiNW and exhibit the efficiency of the electrical characteristics by oxide thickness (t_ox_) variation, dopant concentration, and thickness of the silicon (t_si_) in the nanometer regime, respectively. Coquand et al. [9] have presented a study of channel performance and electrostatic control for Tri-gate nanowires by reducing gate length and channel width dimensions to the optimum limit. The optimization in the dimensions results in a low Subthreshold Slope (SS) and Drain-Induced Barrier lowering (DIBL). The effects of electron confinements on thin gate cross-sections surrounded by SiO_2_ surface roughness have been discussed by Ramayya et al. [10]. The electron mobility was monotonously reduced due to the surface roughness scattering effect, which was dominant.

Cresti et al. [11] have addressed the surface roughness for an electron mobility reduction in GAA and DG nanowires. The surface roughness in the device had been chosen by transfer characteristic parameters, electron density, and low-field electron mobility. Jin et al. [12] have discussed the carrier scattering and backscattering mechanisms. The backscattering decreases the flow of current, electron density, and carrier velocity when the channel’s position varies. The carrier velocity works in the same operation in both carrier scattering and backscattering. The carrier velocity in carrier scattering may increase perhaps increase more in higher order than the backscattering mechanisms.

Numerous studies have been done on the transport characteristics, charge density distributions, and doping concentrations of SiNWs, which have been discussed both empirically and theoretically [13,14,15,16]. The carrier densities for various cross-sections (square, circular, elliptical, and rectangular) using Poisson Schrodinger (PS) and Drift Diffusion (DD) simulations had been investigated with variations in the gate length and channel position being discussed [17]. The source/drain connections were kept wider than the channel to decrease access resistance. The framework for single and double-gate nano MOSFETs using the NEGF coupled mode technique for NWs with six variants had been provided by Svizhenko et al. [18]. The coupled mode effects for nonuniformity of the transverse potential profile greatly impacted multi-gate nanowires. The transport calculations and the electrical properties, such as the transmission coefficient for Si and GaAs with different orientations using NEGF mechanisms, had been discussed elaborately by Luisier et al. [19,20,21].

Using Density Functional Theory (DFT)-based techniques, the electronic characteristics of mechanical and crystalline properties of nanomaterials whose accuracies and efficiencies were reported when it is subjected to the temperature [22,23]. Kumarasinghe et al. [24] have investigated the electronic properties of pure and doped Si nanowires with dimensions up to 10 nm using large-scale Density Functional Theory (DFT) modeling. Three steps are used in modeling: (1) relaxation of the NW unit cell using the DFT method and extraction of the Hamiltonian and overlap matrices, (2) mode space transformation of the imported Hamiltonian matrices, and (3) non-orthogonal NEGF transport calculations using the mode space Hamiltonians, in a way that is self-consistent with the Poisson’s equation.

The Tight Binding (TB) methodology had been used by many researchers to address all the nanowire features that ultimately depend on the electronic structures. All varieties of nanowires and nanotubes (semiconducting, metallic, oxide, and others) were carried over in this method. The majority of research focuses on calculating the sub-band electronic structure of technological semiconductors and its relationship to factors such as shape, composition, and orientation, respectively [25].

Morioka et al. [26] have presented the electronic band structures of rectangular Si NWs using sp^3^d^5^s* tight-binding models. This method considers one excited s* orbital, p orbitals {p_x_, p_y_, and p_z_}, and d orbitals {d_yz_, d_zx_, d_xy_, d_3z_^2^_-r_^2^, and d_x_^2^_-y_^2^}. The x, y, and z coordinate axes are set at [100], [010], and [001], respectively. The part of each atomic orbital typically depends on the nanowire’s width. The symmetrical character of the d_001_ orbital has the same width dependence configuration of p_001_, but the magnitude of orbital d_001_ was found to be lesser, about nanowire based from p_001_.

This work concentrates on designing a novel nanowire-based on GaAs material to use in RF hybrid applications. To improve the I_ON_ and I_OFF_ ratio, various methodologies have been utilized. The usage of high-ƙ dielectric material, such as La_2_O_3_, has shown various enhancements to create an optimal design to be used in RF application setups. This paper has been organized as follows. The basics of NEGF modeling have been discussed in Section 2. The proposed nanowire structure with six variants has been discussed in Section 3. The mathematical modeling of lanthanum oxide with the self-consistent methodology and the division of the 3D problem into 1D transport and 2D Schrodinger equations were investigated in Section 4. Section 5 discusses the lt and analysis of the work. Finally, Section 6 concludes the work and recommends the future aspects.

## 2. Basics of NEGF Modeling

The electronic properties of hydrogen-passivated compound semiconductor nanowires grown in different crystallographic orientations, specifically the band structures, band gaps, and effective electron masses, were discussed previously [27,28,29,30]. Horiguchi et al. [31] have discussed the Silicon nanowire bandgap dependency on the wire width using effective mass theory calculations and using the boundary conditions envelope between wire confinement potential and the barrier height confinement potential’s finiteness. Several authors have presented nanoscale modeling using green’s function, quantum transport modeling, density matrix calculation, and analyzing electronic devices in equilibrium conditions [32,33,34,35].

Seone et al. [36] have proposed the Gate-All-Around (GAA) Si nanowire MOSFET and the impact of current variability on the channel’s surface roughness was analyzed using 3-D real-space non-equilibrium Green’s function. Mazumder et al. [37] have proposed GAA GaAs TFET, which works under the tunneling phenomenon. The maximum I_ON_/I_OFF_ ratio of TFET is achieved by adjusting the few electric gate insulator and GAA TFET channel architecture, which were been investigated to provide the best band-to-band tunneling and potential amplification. Montazeri et al. [38] have demonstrated the band structure for III–V compound semiconductor nanowires using k.p theory calculations. The calculation of the strain is used to determine the particular nanowire structure, and it had been employed using the elastic theory. The resulting calculated strain was called hydrostatic strain, which depends on the proportions of structural dimensions and is independent of the total size. Ren et al. [39] have modeled nanoscale MOSFETs and estimated the scattering and backscattering coefficients using the scattering theory. The critical length and carrier velocity at the source’s end and the channel’s start were identified using transport models. The ballistic, dual-gate nano transistors used for digital applications with a proper choice of the gate oxide thickness and scaling limit down to 10 nm were discussed [40,41]. Several studies have incorporated the operation of the nanowire in a ballistic regime using analytic models [42,43,44,45] and numerical simulations [46,47]. In the simulation study, the density of states, the electron density, and the conduction band energy (E_c_) variations along the position of the channel were investigated [48].

## 3. Design of Proposed Novel GaAs Nanowire

**Figure 1:**
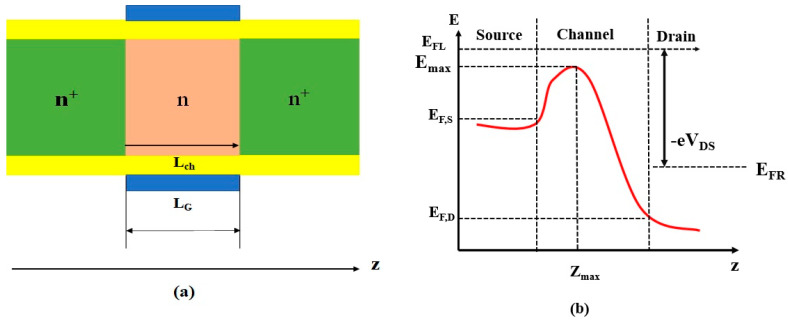
Basic nanowire (**a**) The generic structure where gate length L_G_ equals channel length L_ch_ (**b**) Schematic of the potential energy curve at drain bias V_DS_ in the z-direction.

The generic structure of the proposed nanowire has been designed with a rectangular cross-section with dimensions of 35 nm × 4.5 nm. The source and drain of the Si and GaAs-based nanowire material have a continuous n^+^ donor impurity concentration of (2 × 10^20^ cm^−3^) and n donor doping of (1 × 10^20^ cm^−3^) at the channel. The channel direction in this situation is longitudinal to the <001> *z*-axis, ‘x’ determines the channel width, and ‘y’ determines the current flow into the nanowires, as shown in Figure 1a. The electron movement in the longitudinal z direction is based on Kinetic Energy (E_z_) and is called Transmission Probability T (E_z_). The proposed nanowire dimensions are listed in Table 1.

The Landauer formula, as in reference [3], yields the following description of the drain current:

(1) $$I_{DS}=\frac{e}{\pi h}\sum_{n_{v},n}\int_{0}^{\infty }dE_{z}T(E_{z})\times \left[f(E_{FL},E)-f(E_{FR,}E)\right]$$

(2) $$f(E_{F},E)={\left[1+\exp((E-E_{F})/k_{B}T)\right]}^{-1}$$

(3) $$E=E_{n_{v},n}(z_{\max})+E_{z}$$

where ‘*n*’ is the quantum number that matches the confinement in the wire cross-section; *E_FL_*, and *E_FR_* (*=E_FL_ − eV_DS_*), where *E_FL_* and E_FR_ are the fermi energy levels at the source and drain. Equation (2) is the Fermi-Dirac Distribution. *E_nv_, _n_* in Equation (3) fits into the particular valley *n_v_*, where n_v_ = 1, 2, 3, represents the energy confinement level at the top barrier on the channel as (*E_top_ =E_FL_ + k_B_T*). In Figure 1b, the potential energy distribution is along the *z*-axis, where the maximum energy at the uppermost oxide interface from the channel is represented as E_max_. The Landauer equation can be simplified as follows:

(4) IDS=ekTBπh∑nv,nln(1+exp((EFL−Env,n)/kBT)1+exp((EFR−Env,n)/kBT))

**Figure 2:**
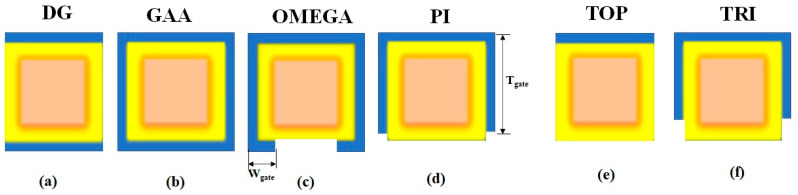
Variants of nanowires (**a**) DG (**b**) GAA (**c**) OMEGA (**d**) PI (**e**) TOP (**f**) TRI.

Equation (4) determines the current *I_DS_* where confinement energy levels at the oxide interface top barrier z_max_ exist. Multiple gates or very thin film structures were necessary to control SCEs in III–V technologies, as suggested previously [3]. There are six variants shown in Figure 2. Each variant differs in the number of gates and their arrangement with natural length, as shown in Table 2.

The natural length λ_n_ can be calculated by:

(5) $$\lambda_{n}=\sqrt{\frac{\varepsilon_{si}}{\tilde{n}\varepsilon_{ox}}\left(1+\frac{\varepsilon_{ox}t_{si}}{4\varepsilon_{si}t_{ox}}\right)t_{si}t_{ox}}$$

where ‘*ñ*’ is referred to as the effective number of gates. The idea was to design devices with both doped and undoped channels that use mid-gap gate material and yields the highest gate efficiencies for sub-10 nm technology [27,28]. The device’s short-channel behavior has been enhanced by raising the equivalent gate number ‘*ñ*’ and by maintaining the size of the gate length (approximately) five to ten times greater than that of the natural length *λ_n_*.

Scaling is possible with GAA devices because they are built with the gate in contact with the channel on all sides. The main benefit of GAA devices is that they have a higher I_ON_/I_OFF_ ratio. Owing to the asymmetric characteristics of the electrostatic control, the tri-gate arrangement results in a lower gate-controlled charge and is 25% smaller when compared to the GAA SiNW for the specific W/H ratio because there are more channel sides placed towards the gate contact.

**Figure 3:**
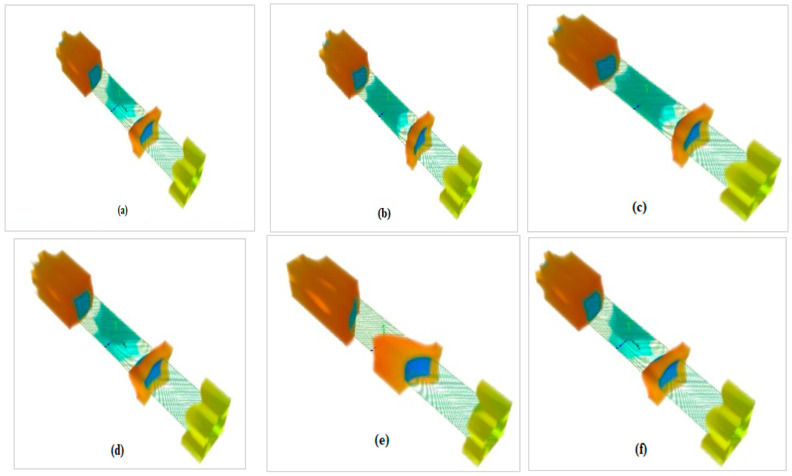
3D potential profile of the silicon nanowire for all variants at V_G_ = 0 V (**a**) DG (**b**) GAA (**c**) OMEGA (**d**) PI (**e**) TOP (**f**) TRI.

When the gate voltage V_G_ = 0 V, the potential in three-dimensional form has been distributed out over the length of the NW, as shown in Figure 3, which is represented in the order of Double gate, GAA, Omega, Pi, Top, and Tri-gate respectively. Three different effective masses (m_l_, m_t_, m_t_), (m_t_, m_l_, m_t_), and (m_t_, m_t_, m_l_) have been considered for the x, y, and z directions. The (m_l_) and (m_t_) are the longitudinal and transverse effective masses whose value is equal to 0.98 m_0_ and 0.19 m_0._ The mass (m_0_) is called free electron mass.

The sub-bands in the z longitudinal direction are more energetic than those in the valley pairs along x and y whose transport mass (m_t_) is less than longitudinal mass (m_l_). To simulate Δ valley for the electrons near the Z [001] direction for Si, the effective masses (m_x_, m_y_, and m_z_) obtained for valleys 1, 2, and 3 are (0.19, 0.19, and 0.98), (0.38, 0.38, and 1.17), and (0.57, 0.57, and 1.36), respectively. Similarly, the effective masses (m_x_, m_y_, and m_z_) for GaAs NW obtained for valleys 1, 2, and 3 are (0.067, 0.067, and 0.542), (0.134, 0.134, and 0.069), and (0.201, 0.201, and 0.676), respectively.

## 4. Mathematical Modeling of the Nanowire with La_2_O_3_

The proposed multi-gate device is a 3-D-dimensional nanowire with a source and drain doping concentration of 2 × 10^20^ cm^3^. The source and drain are made of silicon or gallium arsenide that has been highly doped with n^+^ atoms. The device’s effective mass Hamiltonian has been denoted by the notation:

(6) $$H^{3D}\psi (x,y,z)=E\psi (x,y,z)$$

(7) $$H^{3D}=-\frac{h^{2}}{2m_{x}^{*}}\frac{\partial^{2}}{\partial x^{2}}-\frac{h^{2}}{2}\frac{\partial }{\partial y}\left(\frac{1}{m_{y}^{*}}\frac{\partial }{\partial y}\right)-\frac{h^{2}}{2}\frac{\partial }{\partial z}\left(\frac{1}{m_{z}^{*}}\frac{\partial }{\partial z}\right)+V(x,y,z)$$

where the conduction band edge profile is represented by V (x, y, z) [26], and *m*^∗^*_x_*, *m*^∗^*_y_*, and *m*^∗^*_z_* are the effective masses:

(8) $$V(x,y,z)=E_{C1/2}(x,y)-\phi (x,y,z)$$

where *E_C_*_1/2_ (*x*, *y*) is the band gap of the nanowire core material (Si/GaAs), the point (*x*, *y*) links to the dioxide region, and (*x*, *y*, and *z*) corresponds to space potential. Due to the movement of electrons in the z-direction, the effective core mass in the transport direction is m*_z_, and the effective oxide masses are represented by m*_x_ = m*_x_ (*x*,*y*) and m*_y_ = m*_y_ (*x*,*y*), respectively.

The wavefunction of the three-dimensional Hamiltonian (*x*, *y*, and *z*) in the longitudinal *z* direction is given as:

(9) $$\psi (x,y,z)=\sum_{m}\varphi_{b}(z)\psi_{b}(x,y;z)$$

The *b*th mode eigen function *ψ_b_* (*x*, *y*; *z*) represented in two-dimensional (2D) Schro dinger equation is given as:

(10) $$H^{2D}\psi (x,y;z)=E_{b}(x)\psi_{b}(x,y;z)$$

where

(11) $$H^{2D}=-\frac{h^{2}}{2}\frac{\partial }{\partial x}\left(\frac{1}{m_{x}^{*}(x,y)}\frac{\partial }{\partial y}\right)-\frac{h^{2}}{2}\frac{\partial }{\partial y}\left(\frac{1}{m_{y}^{*}(x,y)}\frac{\partial }{\partial y}\right)+V(x,y;z)$$

(12) $$H^{2D}=-\frac{h^{2}}{2}\frac{\partial }{\partial x}\left(\frac{1}{m_{x}^{*}(x)}\frac{\partial }{\partial x}\right)-\frac{h^{2}}{2}\frac{\partial }{\partial y}\left(\frac{1}{m_{y}^{*}(y)}\frac{\partial }{\partial y}\right)+V(x,y;z)$$

Under boundary conditions, the wave functions at the margins of the two-dimensional (2D) cross-section plane is known as uncoupled mode space method, which eliminates the coupling among several modes (or sub-bands), and *φ_b_* (*z*) satisfies as follows:

(13) $$\left\{-\frac{h^{2}}{2m_{z}^{*}}\frac{\partial^{2}}{\partial z^{2}}+E_{b}(z)\right\}\varphi_{b}(z)=E\varphi_{b}(z)$$

The Schrodinger Equation (13) with open boundary conditions describes the 1-D transport problem, and further, the NEGF technique [31] has been used to solve it. The primary notation for the sub-band b using 1-D Green’s function (*G_b_*) is as follows:

(14) $$G_{b}={\left[E-H_{b}^{1D}-\sum S.b-\sum D.b\right]}^{-1}$$

where

(15) $$H^{1D}=-\frac{h^{2}}{2m_{z}^{*}}\frac{\partial^{2}}{\partial z^{2}}+E_{b}(z)$$

*Σ*_S,*b*_ and *Σ*_D,*b*_ are the S/D self-energies of sub-band *b*, respectively. The 1D charge density *n_k_*^1D^(*z*) in the *b*th sub-band is then obtained via:

(16) $$n_{b}^{1D}(x)=\frac{1}{2\pi \Delta_{x}}\int dEf_{s}G\Gamma_{S.b}G_{b}^{†}+f_{D}G_{k}\Gamma_{D.b}G_{b}^{†}$$

where Δ_x_ is the lattice spacing, and Γ*_S_*_,*b*_ and Γ*_D_*_,*b*_ are defined by:

(17) $$\Gamma_{S.b}=i\left(\sum_{S.b}-\sum_{S,b}^{†}\right),$$

(18) $$\Gamma_{D.b}=i\left(\sum_{D.b}-\sum_{D,b}^{†}\right),$$

The Fermi Distribution functions and the Fermi Energies at the source and drain are given as follows:

(19) $$f_{SD}(E)=\frac{1}{1+e^{\left(E-E_{F}^{SD}\right)/k_{B}T}}$$

The 3-D quantum charge density has been employed in Poisson’s equation after one-dimensional (1D) charge densities of each sub-band are resolve as follows:

(20) $$n^{3D}(x,y,z)={\sum_{k}n_{b}^{1D}(x)\left|\psi_{b}(x,y;z\right|}^{2}$$

(21) $$\nabla^{2}\phi (x,y,z)=-\frac{q}{\in }\left(N_{D}(x,y,z)-n^{3D}(x,y,z)\right)$$

Equation (21) determines the potential and doping profile (*N_D_*) of (*x*, *y*, and *z*). The current in Equation (22) is calculated using the Landauer–Buttiker formula, once self-consistency and charge distributions are attained:

(22) $$I_{D}=2\frac{q}{h}\sum_{b}\int dET_{b}(E)(f_{s}(E)-f_{D}(E)),$$

where the Transmission Probability T_b_ (E) for sub-band ‘b’ is given by:

(23) $$T_{b}(E)=Tr(\Gamma_{S.b}G_{b}\Gamma_{D.b}G_{b}^{†})$$

### 4.1. Numerical Approaches

The two-dimensional (2-D) Schrodinger equations and the 1-D NEGF equation numerical solutions have been presented. The mass discontinuity across the lanthanum oxide (La_2_O_3_) contact has been included in the 2-D Schrodinger equation using the following k-space approach.

#### 4.1.1. K-Space Solutions of Two-Dimensional Schrodinger Equations

Let’s first rewrite Equation (25) as follows:

(24) $$\psi (x,y)=\psi_{b}(x,y;z)$$

where *A_k_*’s are expansion coefficients and |K〉 is a basic set. The eigenvalue problem is solved by substituting Equation (24) in Equation (10) and multiplying 〈*L*\ by the equation sides:

(25) $$\sum_{K}H_{LK}^{2D}A_{K}=E_{b}A_{L}$$

where *H_LK_*^2*D*^*A_K_*_=_ 〈*L*\*H*^2*D*^|*K*〉. In the standard k-space solution [19]:

(26) $$\left|K\rangle =\sqrt{\frac{2}{L_{x}}}\sqrt{\frac{2}{L_{y}}}\sin(k_{i}x)\sin(k_{j}y\right)$$

Here, *L_x_* and *L_y_* are the cross-side section’s lengths in the *x* and *y* directions, respectively.

(27) $$k_{i}=\frac{\pi i}{L_{x}}(i=1,\ldots N_{x})$$

(28) $$k_{j}=\frac{\pi j}{L_{y}}(j=1,\ldots N_{y})$$

The corresponding grid numbers in the *x* and *y* directions are *N_x_* and *N_y_*. It must be noted that the *K* index is derived with the indices *i* and *j* by the formula *K* = (*N_x_* (*i* − 1) + *j*) in Equation (26).

**Figure 4:**
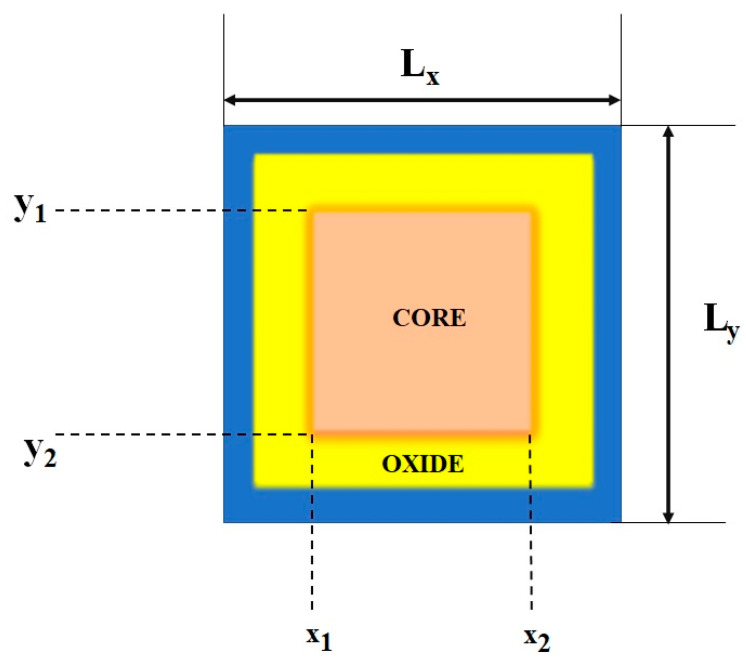
Cross section of a nanowire.

A rectangular cross-section with core/oxide interfaces at (*x*_1_ and *x*_2_) and (*y*_1_ and *y*_2_), respectively (see Figure 4). Equation (29) defines the effective asymmetrical masses at the core/oxide interfaces for the Hamiltonian using *H_LK_*^2*D*^ and it is given as follows:

(29) $$H_{LK}^{2D}=H_{LK}^{0}+H_{LK}^{\left(x\right)}+H_{LK}^{\left(y\right)}$$

where

(30) $$H_{LK}^{0}=\frac{4}{L_{x}L_{y}}\int_{0}^{L_{x}}\int_{0}^{L_{y}}dxdy\sin(k_{u}x)\sin(k_{v}y)\left(\frac{h^{2}k_{i}^{2}}{2m_{x}^{*}(x,y)}+\frac{h^{2}k_{i}^{2}}{2m_{y}^{*}(x,y)}+V(x,y)\right)\times \sin(k_{i}x)\sin(k_{j}y)$$

(31) $$H_{LK}^{\left(x\right)}=\frac{2h^{2}k_{i}}{L_{x}L_{y}}(\sin(k_{u}x_{1})\cos(k_{i}x_{1})-(\sin(k_{u}x_{2})\cos(k_{i}x_{2}))\left(\frac{1}{m_{core,x}^{*}}-\frac{1}{m_{ox}^{*}}\right)\times \int_{y_{1}}^{y_{2}}dy\sin(k_{j}y)\sin(k_{v}y)$$

(32) $$H_{LK}^{\left(y\right)}=\frac{2h^{2}k_{j}}{L_{x}L_{y}}(\sin(k_{u}y_{1})\cos(k_{j}y_{1})-(\sin(k_{v}y_{2})\cos(k_{j}y_{2}))\left(\frac{1}{m_{core,y}^{*}}-\frac{1}{m_{ox}^{*}}\right)\times \int_{x_{1}}^{x_{2}}dx\sin(k_{i}x)\sin(k_{u}x)$$

where $m_{core,x}^{*}$ and $m_{core,y}^{*}$ are the effective core masses in the *x* and *y* directions. The u and v are the indices that are mapped with *L* in a parallel fashion to the index *K,* respectively.

#### 4.1.2. Product Space Solutions of 2-D Schrodinger Equations

From Figure 4, the rectangular cross-section with effective mass is represented as follows:

(33) $$m_{x}^{*}(x,y)=\left\{ \begin{array}{l} m_{x}^{*}(x){\;\mathrm{if}\;y}_{1}\leq y\leq y_{2}\\ m_{ox}^{*}{\;\mathrm{if}\;y\;<\;y}_{1}\;or{\;y\;>\;y}_{2} \end{array}\right.$$

where

(34) $$m_{x}^{*}(x)=\left\{ \begin{array}{l} m_{core,x}^{*}{\;\mathrm{if}\;x}_{1}\leq x\leq x_{2}\\ m_{ox}^{*}{\;\mathrm{if}\;x\;<\;x}_{1}\;or{\;x\;>\;x}_{2} \end{array}\right.\;$$

For a good approximation, it is written as follows:

(35) $$m_{x}^{*}(x,y)=m_{x}^{*}(x)\;\mathrm{for}\;0\leq y\leq L_{y}$$

The oxide region on either side of the core has been considered as (*y*_1_ < *y* < *y*_2_) and the amplitude in the top and bottom of the oxide regions are considered as (*y* < *y*_1_ and *y* > *y*_2_), respectively. The band gap of the oxide materials is substantially wider than that of the core material. Equations (36) and (37) define the effective masses in the y direction:

(36) $$m_{y}^{*}(x,y)=m_{y}^{*}(y)\;\mathrm{for}\;0\leq x\leq L_{x}$$

where

(37) $$m_{y}^{*}(y)=\left\{ \begin{array}{l} m_{core,x}^{*}{\;\mathrm{if}\;y}_{1}\leq y\leq y_{2}\\ m_{ox}^{*}{\;\mathrm{if}\;y\;<\;y}_{1}\;or{\;y\;>\;y}_{2} \end{array}\right.\;$$

The adjacent side of the oxide effective mass regions is inappropriate for the above reasons. Therefore, it is written as under good approximation:

(38) $$H^{2D}=-\frac{h^{2}}{2}\frac{\partial }{\partial x}\left(\frac{1}{m_{x}^{*}(x)}\frac{\partial }{\partial x}\right)-\frac{h^{2}}{2}\frac{\partial }{\partial y}\left(\frac{1}{m_{y}^{*}(y)}\frac{\partial }{\partial y}\right)+V(x,y)$$

The following equation is the product-space solution:

(39) $$|K\rangle =\chi_{i}(x)\xi_{j}(y),$$

Equation (40) determines the 1D Schrodinger equation in the x direction, where *χ*_i_ is the *i*th Eigen function:

(40) $$\left\{-\frac{h^{2}}{2}\frac{\partial }{\partial x}\left(\frac{1}{m_{x}^{*}(x)}\frac{\partial }{\partial x}\right)-\bar{V}(x)\right\}\chi_{i}(x)=\in_{i}\chi_{i}(x)$$

where

(41) $$\bar{V}(x)=\frac{1}{y_{2}-y_{1}}\int_{y_{1}}^{y_{2}}dz\;V(x,y)$$

*ζ_j_*(*y*) is the *j*th eigen function and *V*(*x*,*y*) is the confinement potential for the following 1-D Schrodinger equation in the y-direction:

(42) $$\left\{-\frac{h^{2}}{2}\frac{\partial }{\partial y}\left(\frac{1}{m_{y}^{*}(y)}\frac{\partial }{\partial y}\right)-\bar{V}(y)\right\}\xi_{i}(y)=\in_{i}\zeta_{j}(y)$$

where

(43) $$\bar{V}(x)=\frac{1}{y_{2}-y_{1}}\int_{y_{1}}^{y_{2}}dz\;V(x,y)$$

Substituting Equation (39) in Equation (38) and obtaining Equations (40)–(43).

(44) $$\begin{array}{ll} H^{2D}|K\rangle & =-\xi_{j}(y)\frac{h^{2}}{2}\frac{\partial }{\partial x}\left(\frac{1}{m_{x}^{*}(x)}\frac{\partial \chi_{i}(x)}{\partial x}\right)-\chi_{i}(x)\frac{h^{2}}{2}\frac{\partial }{\partial y}\left(\frac{1}{m_{y}^{*}(y)}\frac{\partial \xi_{j}(y)}{\partial y}\right)+V(x,y)\chi_{i}(x)\xi_{j}(y)\\ & =(\in_{i}+\in_{j}+V(x,y)-\bar{V}(x)-\bar{V}(y))|K\rangle \end{array}$$

By multiplying 〈*L*\ in Equation (44), we obtain:

(45) $$H_{LK}^{2D}=\;\in_{L}\delta_{LK}+\langle L\backslash (V(x,y)-\bar{V}(x)-\bar{V}(y))|K\rangle$$

where

(46) $$\in_{L}=\in_{i}+\in_{j}$$

After resolving the Schrodinger equations, the eigenvalue problem and the product-space solution have been found. Equations (40) and (42) illustrate one dimensional (1-D) version of the k-space solution approach, which was introduced in the previous section and has been employed in the modeling.

## 5. Analysis of the GaAs-Based Nanowire

**Figure 5:**
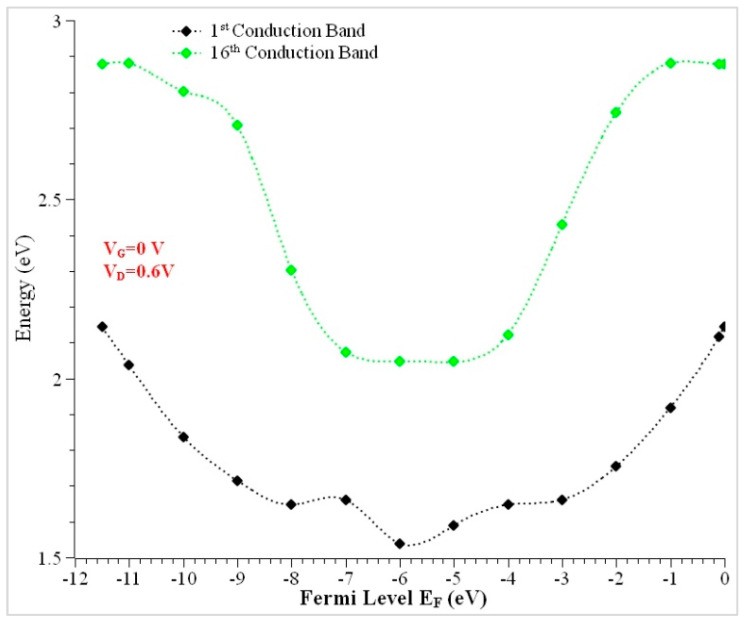
Conduction band energy using the NEGF approach (when V_DS_ = 0.6 V and L_ch_ = 15 nm).

There are six possible structures that have been considered in the simulated NWs. Two distinct materials (Si and GaAs), the Double Gate (DG), Gate-All-Around (GAA), Omega, Pi, Top, and Tri-gate variants have been discussed. Figure 2 depicts the rectangular structure with the physical dimensions of all six variants. The crystallographic orientation z <001> direction has coincided with the channel transport direction. The design parameters of the nanowire, listed in Table 1, have been considered for modeling. The conduction band margins of the NWs for different dielectrics had been addressed previously [49]. Higher gate dielectric constant materials have lower conduction band edges. The SiO_2_ has a greater conduction band edge than La_2_O_3_ when used as a gate dielectric oxide. Thus, lanthanum oxide (La_2_O_3_) has been chosen as a better choice for a gate dielectric oxide and it is one of the best reasons to provide conduction at lower energies. The Si and GaAs rectangular nanowire simulations have been designed with the same wire length (L_wire_ = 35 nm). Figure 5 shows the comparison between the first and last state energy. The first and sixteenth energy levels of conduction band electrons in a rectangular wire differ by 22% at the left contact of the fermi level E_FL_ = −5 eV.

**Figure 6:**
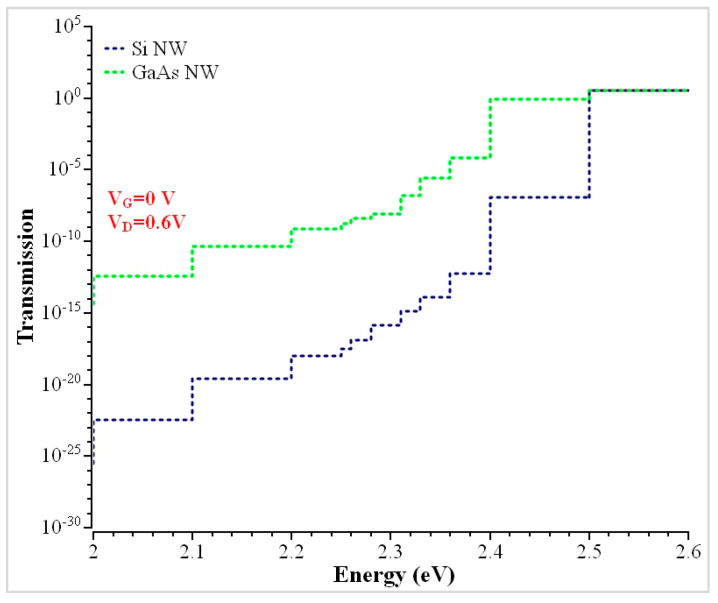
Comparisons of transmission coefficients between Si and GaAs for the 2.5 nm Trigate Nanowire.

Based on the full-band model (sp^3^d^5^s*) model, Figure 6 illustrates transmission coefficients for the 2.5 nm wire in the conduction band. The conduction band reaches high transmission when the thickness of the wire get decreases, as shown previously [50,51].

The ballistic current has been calculated by a comparison of the transmission and energy. The transmission steps depend on the channel and have high transmission regions at an energy E = 2.6 eV. The energy differences are nearly parallel; the higher transmission obtained for both Si and GaAs nanowires are 2.8892 eV and 3.5768 at 2.6 eV. Hence, the GaAs is 1.23 times greater than Si NW. Maximum transmission can be achieved with an increase in wire dimension. Higher transmission had been achieved using different orientations with an increase in gate bias, as shown previously [20]. The transmission spectrum has been fixed with zero gate bias (V_G_) and a drain voltage (V_D_) of 0.6 V. When the gate voltage increases, higher transmission is achieved due to the lowering of the barrier. To normalize the current density in ballistic conditions, the effective width (W_eff_) is assumed to be four times the channel width (W_ch_), as shown previously [11,52].

**Figure 7:**
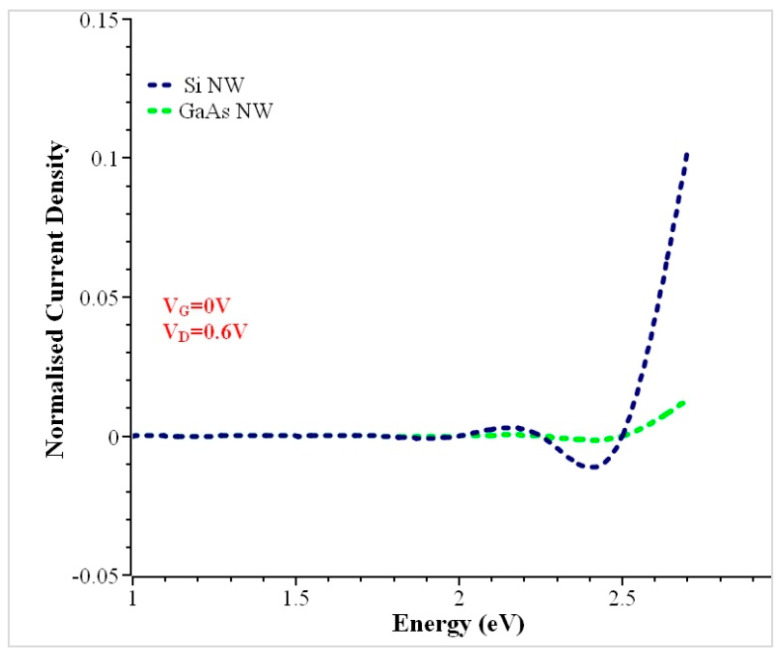
Comparisons of normalized current density for the Si and GaAs Trigate variants.

Figure 7 shows the normalized current density spectra (i_z_/i_z_, avg) calculated by (T × (f_L_ − f_R_)), where T is the transmission and (f_L_ − f_R_) are the left and right fermi level contacts. The normalized current density distribution is uniform in the GaAs NW, and this uniformity occurs when the Wagner number (Wa > 5), as shown previously [53].

**Figure 8:**
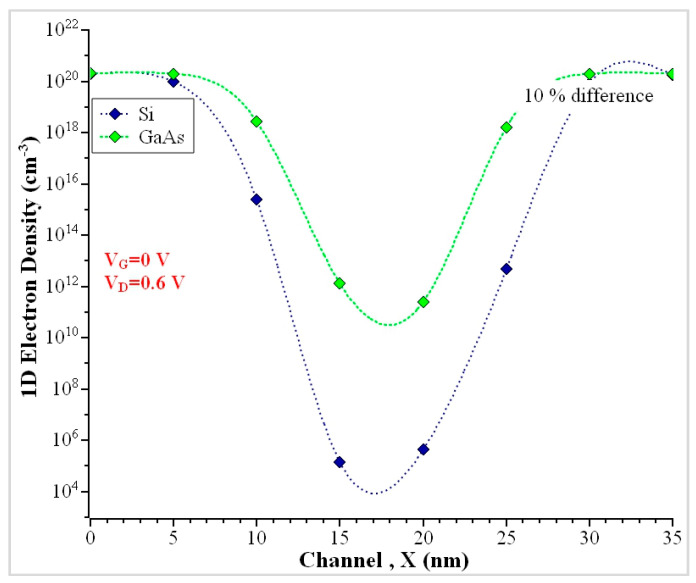
Electron density (N_1D_) along the *Z*-axis.

The one-dimensional electron density (N_1D_) along the channel has been plotted against Si and GaAs NW. The comparison has been noticed specifically at the midchannel ‘z’. The NEGF calculations are made to compute electron density and the electrostatic potential at the interface. At zero gate bias, there is no creation of a potential barrier and electrons to penetrate the channel. The electron density (N_1D_ ~ 1 × 10^20^) cm^−3^ has been obtained in the OFF state when V_G_ = 0 V at the source and decreases more at the midchannel. The electron density increases at the midchannel due to three reasons: (a) Surface Roughness, (b) Higher Gate bias voltage (V_G_ > 0.3 V), or (c) when channel doping is greater than source-drain doping. Here, the middle of the channel has a low electron concentration, which maintains a higher concentration at the drain. The electron densities are uniform throughout the height of the channel, and the GaAs have attained a higher electron concentration at the midchannel than Si NW, as shown in Figure 8.

**Figure 9:**
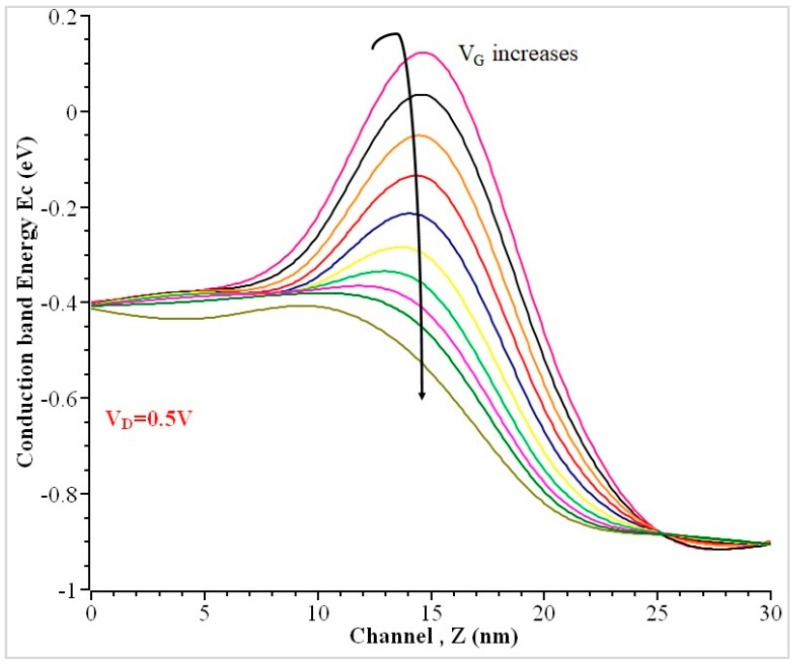
Conduction band energy of the GAA NW along the channel when V_D_ = 0.5 V.

The conduction band energy depends upon the function of both y and z, which is a function of width and length, whereas the sub-band energy minima depends on length [33]. Using the relationship with the carrier velocity, it is concluded that the frequency of electron transmission and channel length are inversely proportional with each other. Thus, the saturation current (I_ON_) increases when the channel length gets reduced. The carriers can travel more easily through shorter gate lengths and channel lengths in comparison to a longer channel, as shown previously [45]. The conduction band edge profiles for the GAA variant by fixing V_D_ = 0.5 V and V_G_ have been varied between 0 and 1, as shown in Figure 9. The device gets off at low gate voltages. When gate voltage increases, the potential barrier gets lower, and the energy attained by the electrons will move faster from source to drain and gets lowered with an increase in drain bias.

**Figure 10:**
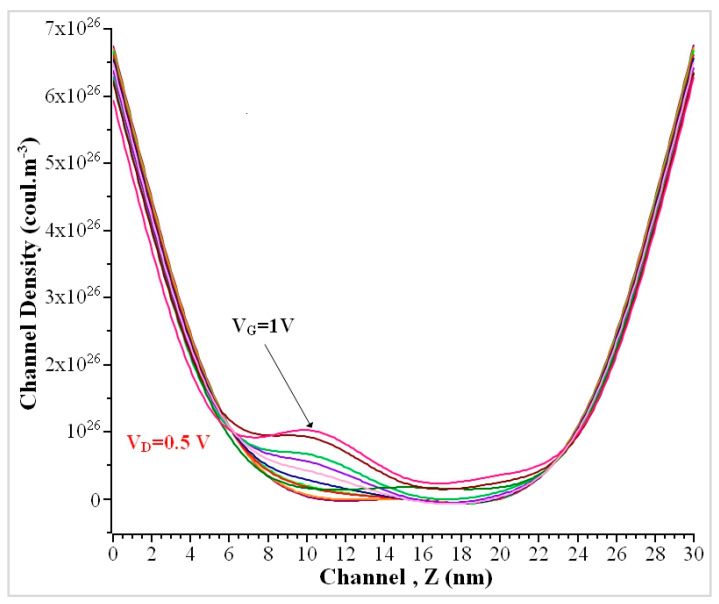
The charge density of the GAA nanowire along the channel when V_D_ = 0.5 V.

The conduction band energy decreases at the midchannel when gate voltage V_G_ increases from 0 to 1 V. Each band energy differs with a voltage of 0.1 V. Due to a higher impurity concentration than that of the channel, a sudden peak charge density (ρ) of 6.75 × 10^26^ Coul.m^−3^ was produced at the source and drain when V_G_ = 0 V. With the increase in gate voltage V_G_ = 0.5 V, the charge density (ρ) over the length of the channel decreases from 6.48 × 10^26^ Coul.m^−3^ to 6.24 × 10^26^ Coul.m^−3^ from the source to the midchannel and then increases when it reaches near the drain terminal, as shown in Figure 10. The charge density increases at the midchannel when gate voltage increases from 0 to 1 V with voltage difference of 0.1 V.

Table 3 shows the comparison modeling results of Si and GaAs Trigate NW. Though the geometrical dimensions are identical for Si and GaAs NW, the accumulation of electrons in GaAs NW is 11% more when it varies with gate voltage when compared to Si NW. Hence, it is evident from the results that the increase in electron density of GaAs NW results in a decrease in current density, which shows that the current density depends upon the property and nature of the material and is independent of electron density. The inversion charge shifts away from the interface in the charge on the quantum modulation effect on Si and GaAs NW, which has been demonstrated to have no impact on the Subthreshold Slope (SS), as shown previously [54]. The log-scale (I_D_-V_GS_) transfer curve with V_GS_ at the subthreshold region has been used to calculate the Subthreshold Slope (SS), which is defined as −[d(log_10_I_D_)/dV_G_].

**Figure 11:**
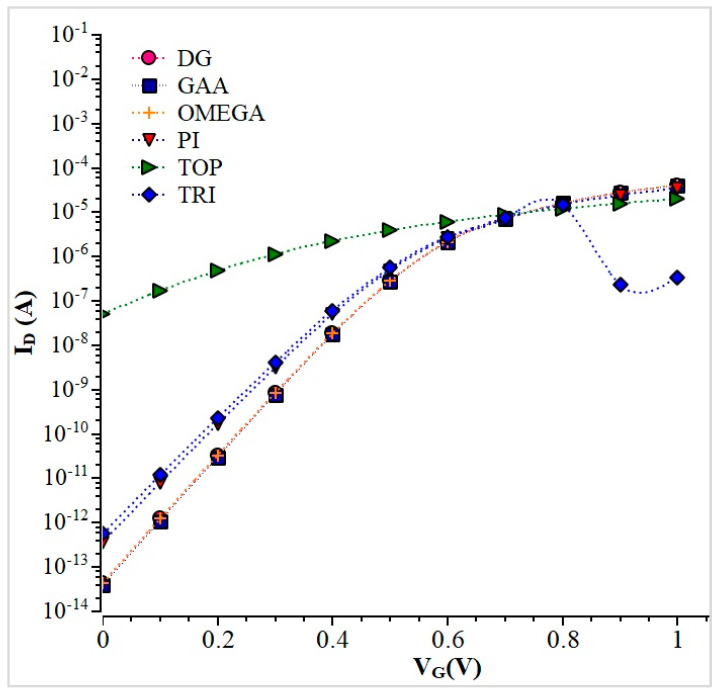
Transfer Characteristics of Si NW.

**Figure 12:**
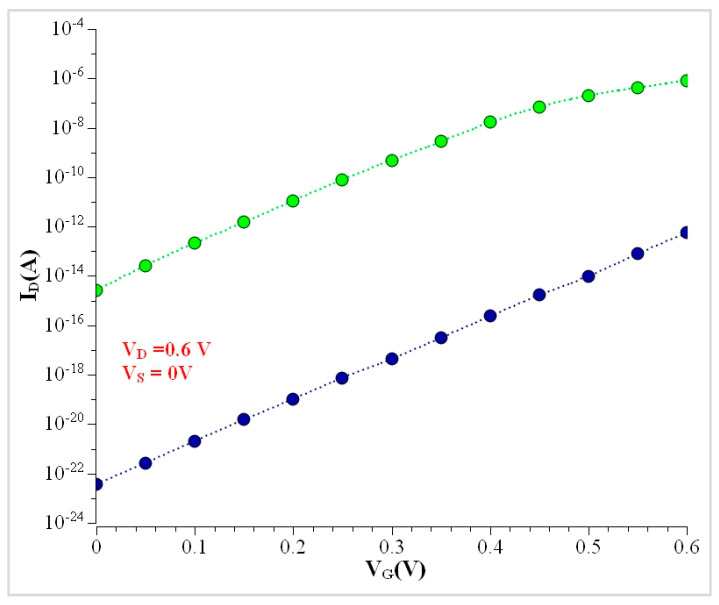
Transfer characteristics comparison for Si and GaAs NWs.

The transfer characteristic curve for Si NW is shown in Figure 11. The GAA device has the highest ON current of 4.09 × 10^−5^ A. Our simulation results of 15 nm Si NW are compared with GaAs NW. Figure 12 shows the transfer characteristics curve for Si and GaAs Tri-gate NW. Here, the I_ON_ current of the GaAs Tri-gate nanowire is 10^−7^ A (V_S_ = 0 V and V_D_ = 0.6 V), and with the silicon nanowire is 10^−13^ A (V_S_ = 0 V and V_D_ = 0.6 V). This analysis shows that the gallium arsenide nanowire, due to its larger I_ON_ current, has more advantages over other types of devices. The results of I_ON_, I_OFF,_ and I_ON_/I_OFF_ are identical values in the simulation results of the Double Gate and Omega variants, as shown clearly in Table 4 comparison results.

It was observed previously [55] that when the gate length increases to 35 nm, the I_ON_/I_OFF_ ratio remains high in GAA NWs compared to all other gates. The Omega and the Double gate NWs modeling results remain the same and high when compared to the Ω -gate MoS_2_FET [56,57], which are shown in Table 4. The GAA has a smaller leakage current than any other gate and a higher conduction band energy of 8% and 37% (at 15 nm) than the Tri-gate and Pi gate. Thus, the GAA Si NW also shows a good Subthreshold Slope (SS) of 176 mV/dec, which is 39% greater than the Tri-gate NW. Therefore, the GAA device has been chosen as a better electrostatic control device.

Our proposed method has been compared with a previous study [11], where channel length (L_ch_ = 15 nm) and oxide thickness (t_ox_ = 1 nm) are the same. Hence, it has been observed that the increase in the gate length (L_G_ ≥ 15 nm) and silicon dioxide material (SiO_2_) material leads to an increase in leakage current. Thus, in our proposed method, the OFF current (I_OFF_) has been reduced by various parameters, such as (1) oxide material with high dielectric constant, (2) gate length scaling, and (3) low threshold voltage.

Table 5 summarizes the Si NW GAA variant with existing SiNW for an S/D doping concentration of 2 × 10^20^ cm^−3^. According to modeling results, the GAA NW has achieved a high I_ON_/I_OFF_ ratio (1.06 × 10^9^) when the width-to-height (W/H) ratio dimensions are equal to 1. For fixed gate and drain voltages, the small (W/H) ratio changes in the geometrical dimensions result in a low I_ON_/I_OFF_ ratio, a high electron density, Subthreshold Slope (SS), and Drain Induced Barrier Lowering (DIBL). The smooth and rough surface in the channel also differs between ON and OFF currents. However, the I_ON_/I_OFF_ ratios are the same in both cases, the smooth surface produces a high ON current (1 × 10^−6^ A) as in Ref. [32].

## 6. Conclusions and Future Recommendations

The electrical characteristics of the NWs shielded with Lanthanum Oxide (La_2_O_3_) material and the orientation with [001] z using the Non-Equilibrium Green Function (NEGF) method were analyzed. Using the NEGF technique, the performance of Silicon and Gallium Arsenide Nanowires with multi-gate structural arrangements, the electrical characteristics, and their parameters are computed. The comparison between all the nanowire variants was simulated. The semi-empirical tight-binding technique (sp^3^d^5^s*) was used to determine the transmission coefficient of Silicon and Gallium Arsenide nanowires for [001] orientations. The transverse dimensions of rectangular nanowires with similar energy levels have been examined, and the comparisons between Silicon and Gallium Arsenide NWs were investigated. The III–V compound semiconductor, such as GaAs NW, shows an attractive simulation in a few parameter results, such as transmission and electron density, compared to Silicon NW. Considering the issue of leakage current reduction, Silicon NWs are more suitable than Gallium Arsenide NWs.

In future work, the comparison between the same wire (Si or GaAs) with different orientations and the same orientations for different materials (Si and GaAs) should be investigated. The problems solved by Gallium Arsenide have focused on III–V compounds along with Silicon or IV–IV compounds, which could be used for applications such as energy storage, flexible electronics, and biomedical devices. Additionally, the development of new synthesis techniques may lead to the production of nanowires with novel compositions and improved properties.

## Figures and Tables

**Table 1:** Physical dimensions.

Device Parameters	Proposed Nanowire
T_ox_ (upper) nm	1
T_ox_(lower) nm	1
Channel Length (L_ch_)	15 nm
Channel Width (W_ch_)	2.5 nm
Channel Height (H_ch_)	2.5 nm
Source Length (L_S_)	10 nm
Drain length (L_D_)	10 nm
S/D n ^+^ Donor doping (cm^−3^)	2 × 10^20^
n channel doping (cm^−3^)	1 × 10^20^
Shape	Rectangular
Transport	100
Confinement and Y direction	010

**Table 2:** Gate variant arrangement.

Gate Variant	No. of Gates *ñ*	Natural Length *λ_n_*
Double Gate	2	1.149
GAA	4	0.8129
Omega Gate	3.4	0.8817
Pi Gate	3.14	0.9175
Top Gate	1	1.625
Tri-gate	3	0.939

**Table 3:** Comparison results of Si and GaAs Tri-gate NW.

Parameter	Si NW	GaAs NW
I_D_max(A)	6.08 × 10^−8^	8.20 × 10^−8^
Transmission	2.889	3.576
Normalized Current Density	1	1.06 × 10^−6^
Electron Density, N_1D_(#/cm^3^)	1.76 × 10^20^	1.98 × 10^20^
I_ON_ (A)	5.6 × 10^−13^	8.21 × 10^−7^
I_OFF_ (A)	3.75 × 10^−23^	2.62 × 10^−15^
I_ON_/I_OFF_	1.49 × 10^10^	3.13 × 10^8^
Subthreshold Slope SS (mV/decade) at V_G_ = 0.1 V to 0.6 V	8 mV to 107 mV	8 mV to 99 mV

**Table 4:** Comparison results of Si NW variants.

Parameter	DG	GAA	OMEGA	PI	TOP	TRI
I_ON_ (A)	4.04 × 10^−5^	4.09 × 10^−5^	4.04 × 10^−5^	3.46 × 10^−5^	1.99 × 10^−5^	3.38 × 10^−7^
I_OFF_ (A)	4.35 × 10^−14^	3.84 × 10^−14^	4.35 × 10^−14^	3.62 × 10^−13^	5.03 × 10^−8^	5.64 × 10^−13^
I_ON_/I_OFF_	0.92 × 10^9^	1.06 × 10^9^	0.92 × 10^9^	0.95 × 10^8^	0.95 × 10^3^	0.6 × 10^6^
Conduction band E_c_ in eV at 15 nm	−0.310	−0.309	−0.310	−0.330	−0.424	−0.334
Charge density ρ (Coul.m^−3^) at 15 nm	6.42 × 10^23^	6.24 × 10^23^	6.42 × 10^23^	1.01 × 10^24^	4.6 × 10^24^	1.01 × 10^24^
Normalized current density	1.03 × 10^29^	1.08 × 10^29^	1.03 × 10^29^	1.06 × 10^29^	1.07 × 10^29^	1.08 × 10^29^
Subthreshold slope (mV)	106	176	106	107	115	108
No. of nodes	9	9	10	10	10	9
No. of iterations	21	21	21	16	16	15
NEGF simulation time in secs	0.277	0.159	0.278	0.250	0.173	0.543
Schrodinger simulation time in secs	0.323	0.333	0.365	0.369	0.348	0.879
Poisson simulation time in secs	0.666	0.582	0.690	0.758	0.711	4.061

**Table 5:** Comparison of various Si nanowires with conventional nanowires.

Nanowire	Oxide (nm)	L_ch_ (nm)	L_G_ (nm)	V_GS_, V_DS_ (V)	I_ON_ (A)	I_OFF_ (A)	I_ON_/I_OFF_
[4]	HFO_2_	5	20	(0.6,0.6)	4.5 × 10^−5^	0.1 × 10^−6^	4500
[6]	SiO_2_	15	5	(1,0.5)	1 × 10^−5^	8.8 × 10^−16^	0.11 × 10^11^
[8]	SiO_2_	11	10	(1,1)	0.87 × 10^−3^	3.4 × 10^−12^	0.25 × 10^9^
[12]	SiO_2_	15	15	(0.3,0.5)	1.22 × 10^−5^	3 × 10^−11^	0.40 × 10^6^
[36]	SiO_2_	6	6	(0.6, 0.001)	1 × 10^−6^	1 × 10^−13^	1 × 10^7^
[49]	La_2_O_3_	10	10	(1,0.5)	5.5 × 10^3^	0.84 × 10^−8^	6.54 × 10^11^
[51]	SiO_2_	5	6	(0.6,0.6)	9.8 × 10^−7^	3.4 × 10^−13^	2.88 × 10^6^
This work	La_2_O_3_	15	2	(1, 0.6)	4.09 × 10^−5^	3.84 × 10^−14^	1.06 × 10^9^
This work	La_2_O_3_	15	2	(0.6, 0.6)	2.11 × 10^−6^	3.84 × 10^−14^	0.55 × 10^8^

## Data Availability

Not applicable.
